# Psychological impact of mass violence depends on affective tone of media content

**DOI:** 10.1371/journal.pone.0213891

**Published:** 2019-04-01

**Authors:** Jolie Baumann Wormwood, Yu-Ru Lin, Spencer K. Lynn, Lisa Feldman Barrett, Karen S. Quigley

**Affiliations:** 1 University of New Hampshire, Department of Psychology, Durham, NH, United States of America; 2 University of Pittsburgh, School of Computing and Information, Pittsburgh, PA, United States of America; 3 Charles River Analytics, Inc., Cambridge, MA, United States of America; 4 Northeastern University, Department of Psychology, Boston, MA, United States of America; 5 Massachusetts General Hospital, Department of Psychiatry and the Athinoula A. Martinos Center for Biomedical Imaging, Charlestown, MA, United States of America; 6 Edith Nourse Rogers Memorial (VA) Medical Center, Center for Healthcare Organization and Implementation Research, Bedford, MA, United States of America; Coventry University, UNITED KINGDOM

## Abstract

Exposure to media coverage of mass violence has been shown to predict poorer mental health symptomology. However, it is unknown whether such media coverage can have ubiquitous effects on average community members, extending to biological and perceptual processes that underlie everyday decision making and behavior. Here, we used a repeated-measures design over the first anniversary of the Boston Marathon bombings to track participants’ self-reported distress, their eye blink startle reactivity while viewing images of the bombings, and their ability to perceptually distinguish armed from unarmed individuals in a behavioral shooting task. We leveraged a computational linguistics method in which we sampled news content from the sources our participants most commonly self-reported reading, and then quantified both the extent of news coverage about the marathon and the affective tone of that news coverage. Results revealed that participants experienced greater current distress, greater physiological reactivity to threats, and poorer perceptual sensitivity when recent news coverage of the marathon contained more affectively negative words. This is the first empirical work to examine relationships between the media’s affective tone in its coverage of mass violence and individuals’ threat perception and physiological threat reactivity.

## Introduction

Over the past decades, it has become apparent that many of the deleterious consequences of mass violence are augmented by widespread media coverage of such incidents. Independent of any direct exposure, greater media exposure to terrorism and mass violence predicts poorer mental health, including greater trauma-related symptoms [1–3] and greater acute stress responses [4, 5], as well as poorer cardiovascular health outcomes [6, 7]. The present investigation for the first time extends these findings beyond mental and physical health symptomology to lab-based behavioral measures of physiological threat reactivity and perceptual threat sensitivity, examining whether the impact of media coverage of mass violence extends to ubiquitous processes underlying everyday decision-making among otherwise healthy adults. In addition, we utilize a novel approach for assessing media coverage, leveraging a computational linguistic method to assess both the quality and quantity of news content related to a recent mass violence incident in the news sources our sample reported reading most frequently. Due to the novelty of this approach, we consider the study to be conducted for the purpose of scientific exploration and discovery (as opposed to being confirmatory in nature).

Previous research on real-world media coverage has also typically focused on the overall extent (or amount) of media coverage with less attention paid to specific features of the media coverage, such as its affective tone, that might increase or decrease the impact of media coverage on biological or psychological outcomes (although see [8, 9]). Empirical work examining the impact of mass violence on threat perception, for instance, has largely been grounded in a theoretical stance suggesting that as threat-relevant information becomes more accessible, individuals will tend to feel they are more likely to encounter threats (e.g., [10–13]). From this perspective, a greater *extent* of exposure to mass violence should produce biased threat-perception regardless of the affective or conceptual features of that exposure (e.g., whether media coverage is focused on the death and devastation caused by a bombing or on the heroics of first responders in the wake of the bombing). Other researchers have argued that emotion is critical in modifying threat perception in response to terrorism (e.g., [8, 14–16]), although this literature has somewhat narrowly focused to date on the perception of terrorism risks or on judgments about terrorism-related events or policies. In one study, however, individuals exposed in the lab to images from a terrorist attack exhibited poorer threat perception sensitivity (i.e., a reduced ability to accurately discriminate threatening from non-threatening stimuli) if the media images to which they were exposed were accompanied by affectively negative headlines (e.g., “Not Since 9/11”) compared to affectively positive headlines (e.g., “Boston Strong”) [17]. These findings demonstrate that the affective or emotional framing of media coverage of an incident of mass violence may critically shape threat perception independent of the extent of coverage. Yet, much remains unknown concerning how media coverage of terrorism, and the affective tone of any such coverage, may shape individuals’ daily threat-related perceptions, physiological reactions, or reported distress.

To fill this gap, in the present repeated-measures study, we assessed changes in self-reported distress, physiological threat reactivity, and threat perception in the context of predictable, naturalistic changes in everyday news content relevant to a terrorist incident. Specifically, we tracked a sample of Boston area residents with three waves of data collection surrounding the first anniversary of the Boston Marathon bombings of April 15, 2013. We predicted (and accurately so) that media coverage of the marathon and the terrorist attack at the marathon the previous year would increase for a short period of time ahead of the next annual running of the Boston Marathon in 2014. At each of the three waves of data collection (several months before, just before, and several months after the 2014 Boston Marathon), participants came to the lab and self-reported their current distress related to the bombings and completed an acoustic startle reactivity task while viewing images taken from media coverage of the Boston Marathon bombings to assess their physiological threat reactivity. They also completed a behavioral shooting task meant to assess threat perception sensitivity (i.e., their ability to perceptually discriminate armed and unarmed individuals) and threat response bias (i.e., their tendency to favor the “shoot” response regardless of what stimulus was shown). Based on past research [17, 18], we predicted that the affective tone of media coverage related to the marathon would predict participants’ symptoms of distress, their physiological threat reactivity, and their threat perception sensitivity (but not their threat response bias; see [17]) independent of the *extent* of media coverage related to the marathon. Specifically, we predicted that when recent media coverage related to the marathon included more affectively negative words, participants would report increased self-reported distress related to the bombings, increased eye blink startle reactivity to images of the bombings, and decreased threat perception sensitivity in the behavioral shooting task compared to when such media coverage included more affectively positive words. Although incidents of mass violence are inherently negative and people need to be made aware of such threats, the present study addresses whether gratuitous negativity, perhaps to increase media viewership or readership, can have unintended adverse impacts on reports of distress, physiological reactivity, perception, and behavior.

## Materials and methods

### Participants

This study was approved by Northeastern University’s institutional review board (Approved Protocol #13-05-04). All participants provided written, informed consent after the nature and possible consequences of the study were explained. Ninety-five participants (36 males, 58 females, 1 undeclared) ages 17–61 (M_age_ = 28.29, SD_age_ = 10.58) from Northeastern University and the surrounding Boston community were recruited for a study on threat perception and the Boston Marathon bombings via advertisements on fliers posted around the city, on Craigslist.com, and in the Boston Metro newspaper. It was made clear in the advertisements that no direct exposure to the Boston Marathon bombings in 2013 was required to be eligible to participate. Given the lack of effect size on which to base an *a priori* power analysis, a target sample size of *N* = 100 was selected based on simulation studies for identifying medium effect sizes in multilevel models [19]. In multilevel designs, the upper-level (between-subject) sample size has more impact on the power than the lower-level (within-subject) sample size (i.e., number of participants affects power more than number of waves; [19, 20]), and simulation studies suggest that estimates of regression coefficients, standard errors, and variance components in multilevel models are generally accurate and unbiased with an upper-level sample size of 100 for slopes of medium effect size [19].

Potential participants completed the 8-item Patient Health Questionnaire (PHQ-8; [21]) and those without significant depressive symptomology (< 10 on the PHQ-8) were eligible to participate. One additional participant consented, but became syncopal during placement of the facial electromyography (fEMG) electrodes for recording startle reactivity and withdrew from the experiment; no data was retained. Following this occurrence (after the first 30 participants had completed the first Wave), potential participants were additionally screened for any history of fainting in medical settings and all were offered a snack prior to beginning electrode placement at each session; no additional such episodes occurred. Of the final sample, 57.9% identified as White, 13.7% identified as Black, 9.5% identified as Asian, 1.1% identified as Pacific Islander, 8.4% identified as more than one race, 5.3% identified as a race not listed, and 3.2% did not report their race. In addition, 12.6% identified as Hispanic, and 60.6% had some form of postsecondary education beyond high school. Thus, we were successful at recruiting a relatively diverse and representative sample from the Boston community.

These 95 participants completed the first wave of data collection (Wave 1) several months before the first anniversary of the Boston Marathon bombings, between February 2, 2014, and March 12, 2014. Ninety-two of these participants (96.8%) returned to complete the second wave of data collection (Wave 2) between March 31, 2014, and April 19, 2014 (in the weeks immediately leading up to the Boston Marathon), and 85 of the original participants (89.5%) returned to complete the third wave of data collection (Wave 3) several months after the anniversary, between September 10, 2014, and November 21, 2014 (with the exception of two of these 85 participants who instead completed it in June 2014 due to Fall conflicts). Participants received $30 for completing Wave 1, $40 for completing Wave 2, and $50 for completing Wave 3. Four participants were excluded from the sample prior to data analyses due to repeated failure to comply with experimental protocols across waves, leaving a final sample of 91 participants. Because analyses were completed using hierarchical linear modeling, which allows for missing data, participants who did not complete all three waves of data collection were retained in the sample for analyses.

### Tasks and measures

#### Marathon recall survey

Participants were first asked to complete a Marathon Recall Survey [17, 22]. The survey comprised 19 open-ended questions and two multiple-choice questions about participants’ feelings and experiences during the week of the Boston Marathon bombings and the subsequent manhunt and citywide lockdown on April 18–19, 2013. This survey was used to elicit thoughts and feelings relevant to the Boston Marathon bombings at the beginning of each experimental session. In so doing, we intended to standardize the timing of participants’ most recent exposure to the bombings across all participants and waves because we reasoned that variability in the timing and extent of the most recent acute exposure would likely impact our dependent measures above and beyond any potential changes associated with the much more diffuse and prolonged changes in media coverage with which this study is primarily concerned. The retrospective self-report data provided on this survey were not analyzed as part of the present investigation.

#### Acoustic startle task

We assessed participants’ physiological reactivity to an aversive (threat-related) stimulus by measuring the amplitude of the startle blink reflex in an acoustic startle paradigm. The startle blink reflex is potentiated by aversive states or contexts (for a review, see [23]). In our task, participants listened to a series of 24 white noise bursts (~95 dB, 50 ms, instantaneous rise time) over binaural headphones while electromyographic (EMG) activity was recorded over the *orbicularis oculi* muscle region under the left eye. These white noise bursts, called startle probes, occurred while the participant viewed images on a computer. They viewed 28 images in a random order: 14 neutral images from the International Affective Picture Set (IAPS; [24]) and 14 images taken from media coverage of the Boston Marathon bombings (e.g., from the *Boston Globe*, the *Boston Metro*, or the *New York Times*). All images were displayed for 6 s with a jittered inter-image interval of 10–16 s during which a white fixation cross was displayed in the middle of a black screen. To reduce the predictability of startle probe presentations, two images of each type were presented without a startle probe, and startle probes were presented at quasi-random intervals following image onset (3–5 s after image onset) on the remaining 24 trials. Half of the images of each type used at Wave 1 were replaced with novel images of the same type at Wave 2, as past research suggested this may improve the reliability of measures of affective modulation of startle [25]. However, we found no differences in the reliability of reactivity measures calculated using novel *vs*. familiar images following Wave 2, and thus we retained the same images in this task from Wave 2 to Wave 3.

#### Shooter bias task

Participants completed a modified version of the Shooter Bias Task developed by Correll and colleagues [26, 27]; all stimuli are described in detail in Correll et al. [26]. Visual noise was added to these original images to increase the difficulty of the task and allow for consistent display times across trials and individuals (as described in [17, 18]). In each trial, participants were shown 1–4 background scenes (e.g., parks, subway stations, street corners), each for a randomly chosen duration between 500 and 1000 ms. The final image of each trial (the target image) was a repeat of the final background scene but contained a person. To the participant, this looked like a person suddenly appeared in the final background scene. Each target person was a white male holding either a gun or a non-threatening everyday object (e.g., camera, wallet). The target image was shown for 500 ms followed by a backward mask. Participants were instructed to respond once the backwards mask appeared, and there was no time limit for the response. Participants responded on each trial by pressing one of two keys on a keyboard, one key to “shoot” at any targets they believed were armed and one key to “not shoot” at any targets they believed were unarmed. There were 40 trials of the task (10 target individuals each shown 4 times: twice armed and twice unarmed). Participants also completed 10 practice trials at each wave to familiarize themselves with the instructions and controls prior to completing the critical trials of the task.

#### Questionnaires

*Demographic information.* At Wave 1 only, participants completed a questionnaire that assessed their age, gender, ethnicity, race, and education level.

*Current distress related to the bombings: Impact of Event Scale (IES).* The IES is a 15-item self-report measure that assesses current subjective distress caused by potentially traumatic events [28]. At each wave of data collection, participants were asked to indicate how frequently a series of statements were true of their thoughts and feelings about the Boston Marathon bombings over the past month, on a 4-point Likert scale from “Not at All” to “Often”. Sample items include “I thought about the Boston Marathon bombings when I didn’t mean to” and “I had waves of strong feeling about the Boston Marathon bombings.” A total current distress score for each wave was calculated by summing responses for all 15 items.

*Media usage questionnaire.* At each wave of data collection, participants were asked to list all media sources that they used over the past two weeks to obtain news, including any newspapers (online and print), television shows, social media sites, and radio programs. For each source they listed, participants also rated the frequency with which they used that news source over the past two weeks on a 5-point scale, where 1 = “Only once”; 2 = “A few times”; 3 = “Most days”; 4 = “Every day”; and 5 = “Multiple times per day”.

*Additional questionnaires*. Participants also completed a number of additional questionnaires unrelated to the current investigation, including measures of self-reported physical symptoms, depressive symptoms, anxiety symptoms, and neuroticism (as described in [29]).

### Procedure

Participants were told that the researchers were interested in whether the Boston Marathon bombings influenced threat perception and that participants would be asked to complete a series of threat perception tasks three times over approximately a 9-month period so that we could assess whether any impact of the bombings on threat perception changed over time. At each wave of data collection, participants came to our laboratory at Northeastern University and completed a nearly identical experiment. After providing informed consent (Wave 1 only), participants first completed the Marathon Recall Survey on a computer. They were then offered the option of taking a short break to have a snack (all except the first 30 participants of Wave 1) to minimize possible symptoms of lightheadedness during fEMG electrode placement. Following this, fEMG electrodes were placed as described above and participants completed the Acoustic Startle Task. The fEMG electrodes were then removed and participants completed the Shooter Bias Task. Finally, participants completed a set of questionnaires at the end of each experimental session. Participants were debriefed in-person at the end of their third experimental session or were sent a debriefing statement via e-mail if they did not complete Wave 3.

### Data processing

#### Physiological data acquisition and processing

To assess the amplitude of the reflexive startle blink response, activity over the orbicularis oculi muscle region was assessed using facial electromyography (fEMG). Two reusable Ag/AgCl electrodes from Mindware Technologies LTD (Gahanna, OH) were filled with a conductive electrolyte gel (Signal Gel from BioMedical Instruments; Warren, MI) and placed over the orbicularis oculi muscle region under the participants’ left eye with a reference electrode in the middle of the forehead. To ensure good signal quality, each site was cleaned with alcohol before electrode placement and the area for the reference electrode placement was also exfoliated using a semi-abrasive lotion (LemonPrep, Mavidon Medical Products). Muscle activity was sampled at 1000 Hz using BioLab v. 3.0.13 (Mindware Technologies LTD; Gahanna, OH), using BioLab’s default EMG filter (gain of 5000, low cutoff of 20 Hz, and high cutoff of 200 Hz). Mindware’s EMG scoring software (v3.1.0I) was used to process the fEMG signal whereby the rectified raw fEMG signal was smoothed with a rolling filter (*f*_*c*_ = 16 Hz). The mean amplitude (in microvolts) of the processed signal in the 40 ms before each startle probe was taken as a baseline measure of muscle activity for that trial. To calculate the startle blink amplitude for each trial, this baseline value was subtracted from the peak amplitude of the processed signal from 0 ms to 200 ms following the presentation of the startle probe. All raw data was subject to visual inspection by trained scorers who identified individual trials for exclusion from analyses, including trials with blink activity during the baseline period, trials with multiple blinks following the presentation of the startle probe, trials with blinks within 0–40 ms after presentation of the startle probe (which is too early to be the reflexive startle blink), or trials with unusual signal characteristics (e.g., movement artifact or signal noise caused by improper electrode placement or skin preparation). Participants who did not have at least 12 scoreable trials were removed from analyses involving this task. Useable startle data was obtained from 80 participants at Wave 1 (84.2%), 86 participants at Wave 2 (93.5%), and 79 participants at Wave 3 (92.9%). For these remaining participants, trial-by-trial data exclusions due to the aforementioned criteria accounted for 2.2% of all data at Wave 1, 2.6% of Wave 2 data, and 3.7% of Wave 3 data.

Although a typical affective modulation of startle paradigm involves comparing the startle blink amplitudes during negative images to startle blink amplitudes during more neutral images (see [30]), this modulation paradigm relies on comparing startle responses within affectively potent and affectively neutral contexts. However, our entire startle task was an affectively potent context: participants were anticipating seeing additional bombing-related imagery throughout the task, even on trials with “neutral” images, and the task was completed only moments after the Marathon Recall Survey, in which participants had reflected on details about their whereabouts, experiences, and feelings during the Boston Marathon bombings and subsequent manhunt of April 2013. Not surprisingly, we found little evidence of image-specific modulation in our task: startle amplitudes did not differ between bombing and neutral images at Wave 1 (*M*_*bomb*_ = 33.69, *SD*_*bomb*_ = 30.29; *M*_*neut*_ = 33.72, *SD*_*neut*_ = 28.97; *t*(79) = 0.04, *p* = .969), Wave 2 (*M*_*bomb*_ = 20.14, *SD*_*bomb*_ = 20.41; *M*_*neut*_ = 21.07, *SD*_*neut*_ = 20.85; *t*(85) = 0.77, *p* = .446), or Wave 3 (*M*_*bomb*_ = 27.33, *SD*_*bomb*_ = 26.29, *M*_*neut*_ = 27.59, *SD*_*neut*_ = 26.94; *t*(78) = 0.37, *p* = .711). Thus, we calculated the mean startle amplitude across all image types for each participant at each wave to use as our measure of physiological threat reactivity. For an analysis that examined differences in modulation of startle by picture type within this overarching affectively evocative context, see [29].

#### Behavioral threat perception measures

Performance on the Shooter Bias Task was analyzed using Signal Detection Theory [31]. For each participant, we calculated two performance parameters: bias and sensitivity. Bias (*c*) is a measure of a participant’s tendency to choose the “shoot” response regardless of the stimulus shown. Bias was calculated as *c = -0*.*5(zH+zF)*, where *zH* and *zF* represent the inverse of the standard normal cumulative distribution for the hit rate and false alarm rate, respectively. Sensitivity (*d’*) is a measure of a participant’s ability to discriminate between armed and unarmed targets, and was calculated as *d’ = zH-zF*. To avoid the infinite z-scores that occur when a participant has a false alarm rate of 0 or a hit rate of 1, we used a procedure recommended by Wickens [31] and utilized in similar prior work (e.g., [17, 18, 32]) and set a minimum false alarm rate of 1/(*n*+1) and a maximum hit rate of 1-(1/(*n*+1)), where *n* represents the number of valid object or gun trials, respectively. Because sensitivity and bias tended to be strongly related in our sample, we controlled for sensitivity in analyses involving bias and vice versa. Shooter bias data for one participant at Wave 1 and one participant at Wave 2 were excluded because they were outliers on sensitivity (>3 *SD* below mean).

#### Time- and content-dependent measures of news media coverage

To assess the impact of news media coverage over the first anniversary of the Boston Marathon bombings, we first identified the four most frequent news outlets that participants reported using on the Media Usage Questionnaire at Waves 1 and 2: *The Metro (MT)*, *The New York Times (NY)*, *The Boston Globe (BG)*, and *The Boston Herald (BH)*. Other outlets, such as *USA Today* and the *Wall Street Journal*, were each reported by fewer than 10 respondents. Therefore, our data collection focused on the news published by the four most frequently reported outlets. We used Google News, a news aggregator website, to retrieve news articles published by the four outlets on a daily basis. We first collected the URLs of the news articles from Google News, and retrieved and parsed the news content using the Java HTML parser, jsoup (jsoup.org). The data were drawn from a period of about two weeks before each Wave of data collection began until the end of each Wave of data collection. S1 Table in the supplemental online materials shows the durations of each wave, the duration of media data collection for each wave, and a summary of the media data collected. In total, we collected over 38.5K news articles covering the three waves of the study.

From the collected news articles, we first isolated news articles relevant to our study. We defined marathon-related news as any articles using the keyword “marathon”, which were assumed to be relevant to the 2014 Boston Marathon and/or the bombings that occurred at the 2013 Boston Marathon. As an alternative strategy, we also attempted to isolate only news articles that contained both the keyword “marathon” as well as at least one of the keywords “terror” or “bomb” or any iteration thereof (e.g., terrorist, terrorists, terrorism, bombing, etc.). However, articles identified with this criterion were deemed too sparse for analyses. The number of marathon-related articles for each outlet at each wave can be found in S1 Table in the supplemental online materials. We focused on these marathon-related articles to derive time- and content-dependent measures of news media coverage.

To measure the *affective tone of recent marathon-related media coverage* for each participant at each Wave, we first identified the number of words with positive and negative connotations in each article. Identification of affective words and the classification of affective words as negative or positive was determined using the psycholinguistic lexicon Linguistic Inquiry and Word Count (LIWC; [33]). We then calculated a sentiment ratio (SR): the ratio of the number of positive words (P) to the total number of positive (P) and negative words (N) for each article (*SR = P/(P+N)*). We did not use the simpler ratio P/N because this ratio could be arbitrarily large, which can result in misleading analyses/models. We then took the average of this sentiment ratio from all news articles identified as marathon-related and published on each day *t* from each outlet, where this daily average for each outlet is given by: *SR*_*O*_(*t*). To address data sparseness, we then employed a rolling average for this variable, creating a smoothed variable, ${\tilde{SR}}_{O}(t)$, such that: ${\tilde{SR}}_{O}(t)=\frac{1}{\delta }\sum_{t-\delta <t^{\prime }<t}{\tilde{SR}}_{O}(t\prime )$, where δ is chosen to be 7 days in this study and *t’* is a running variable indicating the range of the smoothing (from *t- δ* to *t*, for each *t*). Next, the smoothed variable, ${\tilde{SR}}_{O}(t)$, was re-scaled from -1 to 1, where -1 indicates that, over the time period δ (i.e., 7 days), all affective words in marathon-related articles were negative, 1 indicates that all affective words in marathon-related articles were positive, and 0 indicates an equivalent number of positive and negative affective words. Plots of ${\tilde{SR}}_{O}(t)$, for each outlet, for each wave can be found in the supplemental online materials (S1 Fig). Finally, to capture whether marathon-related news coverage was more affectively positive (v. affectively negative) *for each participant at each wave*, we took the average of ${\tilde{SR}}_{O}(t)$ across all four outlets over only the 14 days prior to each participant’s in-lab sessions. Thus, for each wave (*w*), each participant (*i*) had a single variable (*X*_*wi*_) that reflected the affective tone (i.e., the positive v. negative affective word usage) in recent news coverage (i.e., in only articles published within the two weeks prior to their in-lab session within each wave) from only the news articles identified as marathon-related.

Next, to measure *the extent of recent marathon-related media coverage* for each participant at each Wave, we first calculated a "relevance rate" (*RR*) for each outlet, capturing the extent to which the news articles from a particular outlet covered marathon-related content. The variable *RR*_*O*_(*t*) indicates whether a given outlet (o) published at least one marathon-related article on a particular day *t*. Once again, to address data sparseness, we employed an identical rolling average for this media variable, creating a smoothed variable, ${\tilde{RR}}_{O}(t)$, such that: ${\tilde{RR}}_{O}(t)=\frac{1}{\delta }\sum_{t-\delta <t^{\prime }<t}{\tilde{RR}}_{O}(t\prime )$, where δ is chosen to be 7 days in this study and *tʹ* is a running variable indicating the range of the smoothing (from *t- δ* to *t*, for each *t*). Values of ${\tilde{RR}}_{O}(t)$ range from 0 to 1 and indicate the likelihood that readers of these newspapers had the chance of seeing at least one marathon-related news article every day over the time period δ (i.e., 7 days). Plots of ${\tilde{RR}}_{O}(t)$, for each outlet, for each wave can be found in the supplemental online materials (S1 Fig). Finally, to capture the extent of marathon-related news coverage *for each participant at each wave*, we took the average of ${\tilde{RR}}_{O}(t)$ across all four outlets over only the 14 days prior to each participant’s in-lab sessions. Thus, for each wave (*w*), each participant (*i*) had a single variable (Z_*wi*_, which ranged from 0 to 1) that reflected the likelihood that readers of these newspapers would see at least one marathon-related news article every day over the two weeks prior to participant (*i*)’s in-lab session within wave (*w*).

These media variables were selected to balance competing concerns about objectivity and specificity. We focused on specific outlets that our sample reported reading in general and focused on only articles in each outlet from the two weeks prior to each participants’ in-lab session at each wave. However, we opted not to assess participants’ potentially biased memory for which specific content they read over the past two weeks, nor their perceptions of the affective tone of the articles they remembered reading. Thus, the two media variables derived here are meant to assess media coverage more generally, not media *exposure*. Our measures are indices of the content and affective tone of written news media coverage more generally during each wave of our study, such that the average content and tone in these four sources (which the vast majority of our sample reported reading) are being taken as *representative of* the average content and tone of written news media in the two weeks immediately preceding each participants’ three in-lab sessions.

### Analyses

The primary purpose of the present study is to examine whether naturally occurring increases/decreases in the amount and affective tone of media coverage of a recent terrorist event are associated with changes in threat perception, physiological reactivity, and self-reported distress related to the event. The repeated-measures design of our study was selected to allow us to collect self-report, physiological, and behavioral measures at time points with varying media coverage of the event. We focused our study around the first anniversary of the Boston Marathon bombings of 2013 because we expected it would allow us to capture threat perception at times when we predicted media coverage of the bombings would be fairly infrequent (i.e., at waves 1 and 3, several months removed from the anniversary) and at a time when we predicted media coverage would be more frequent (i.e., at wave 2, in the weeks immediately leading up to the anniversary and the next running of the annual Boston marathon). The repeated-measures design also allows us to control for potentially large individual differences in our self-report, physiological, and behavioral outcome measures not associated with changes in media coverage of the marathon because it enables us to examine *within-person changes* in these measures across times when the amount and tone of media coverage of the event differ.

To examine whether changes in the extent or affective tone of recent marathon-related news coverage predicts corresponding changes in threat perception or physiological threat reactivity, we analyzed our data using hierarchical linear modeling (HLM; [34, 35]). HLM allows us to model variability across waves nested within each participant (i.e., to examine within-person changes across waves). This approach has advantages over traditional methods of analyzing repeated-measures data (e.g., repeated-measures ANOVA), including simultaneous estimation of within-subject and between-subject variance, more efficient estimation of effects, and lower Type-1 error rates [36]. For our primary analyses, all models were of the general form:

$${\hat{Y}}_{wi}=\pi_{0i}+\pi_{1i}(X_{wi})+e_{wi}$$

where

$$\pi_{0i}=B_{00}+r_{0i}$$

and

$$\pi_{1i}=B_{10}+r_{1i}$$

where *Y*_*wi*_ refers to one of the four outcome variables (i.e., threat perception bias, threat perception sensitivity, physiological threat reactivity as measured by startle amplitude, or current distress related to the bombings) collected at the in-lab session for wave (*w*) nested within participant (*i*); *X*_*wi*_ refers to the affective tone of recent marathon-related news coverage to which participant (*i*) was likely exposed in the two weeks prior to their in-lab session within wave (*w*); *e*_*wi*_ refers to the wave-level error, *B*_*00*_ and *B*_*10*_ refer, respectively, to population-level estimates for the intercept and slope linking the affective tone of recent marathon-related news coverage to the outcome variable; and *r*_*0i*_ and *r*_*1i*_ refer, respectively, to the participant-level variability in the intercept and slope values (i.e., across-participant variability). In addition, as mentioned above, threat perception bias was controlled for in all analyses involving threat perception sensitivity and vice versa due to their strong correlation in the present sample. Similar analyses were also then performed to analyze the influence of the *extent* of recent marathon-related news coverage on all four outcome variables (by replacing *X*_*wi*_ with *Z*_*wi*_ in the generic model described above).

These analyses were completed using the HLM 7 software package for hierarchical linear and nonlinear modeling from Scientific Software International, Inc. [34], utilizing a continuous sampling model with a restricted maximum likelihood method of estimation for model parameters and parameter estimates calculated with robust standard errors. Following the recommendation of [37], for all analyses, all Level-1 variables (i.e., within-person variables such as startle amplitude or affective tone of recent marathon-related news coverage) were centered around each participant’s own across-wave mean.

The HLM analyses described here provide information on the average within-person relationships between the extent (or affective tone) of recent media coverage on the marathon and each of the four dependent variables. The models allow each of these relationships to vary across participants but do not take into account time or the order in which the various data points were collected. For instance, although the amount of self-reported distress might be highest at wave 1 for some participants, it was highest at wave 2 for other participants, and highest at wave 3 for still others. The analyses described here examine whether higher/lower values of the extent (or affective tone) of recent media coverage are associated with higher/lower values of the four dependent variables *within individuals*, and thus, is independent of the chronological ordering of the data.

## Results

Estimated means and standard errors for all variables by wave of data collection are provided in Table 1. However, as described above, our primary analyses focus on modeling within-person effects as opposed to sample-level changes over time.

**Table 1:** Estimated means and standard errors by wave.

Variable	Wave 1	Wave 2	Wave 3
Self-reported Distress	10.69 (1.06)^a^	9.17 (1.06)	7.21 (1.07)^a^
Startle Amplitude	33.92 (3.26)^a^	20.74 (2.12)^a^	27.33 (2.95)
Perceptual Sensitivity (*d’*)	0.55 (.05)^a^	0.68 (0.05)^a^	0.66 (0.05)
Threat Response Bias (*c*)	0.19 (0.04)^a^	0.05 (0.05)^a,b^	0.21 (0.05)^b^
Extent of Marathon-related Media Content (Zwi)	0.23 (0.01)^a^	0.49 (0.003)^a^	0.41 (0.01)^a^
Affective Tone of Marathon-related Media Content (Xwi)	0.04 (0.004)^a^	0.10 (0.001)^a^	0.12 (0.003)^a^

Note: A slopes-as-intercepts approach in HLM was used to generate estimated means and standard errors (given in parentheses) for each variable across waves. Significant differences (α = .05, two-tailed) were determined using chi-square comparisons and are indicated by shared superscripts.

### Affective tone of recent marathon-related media coverage

**Fig 1:**
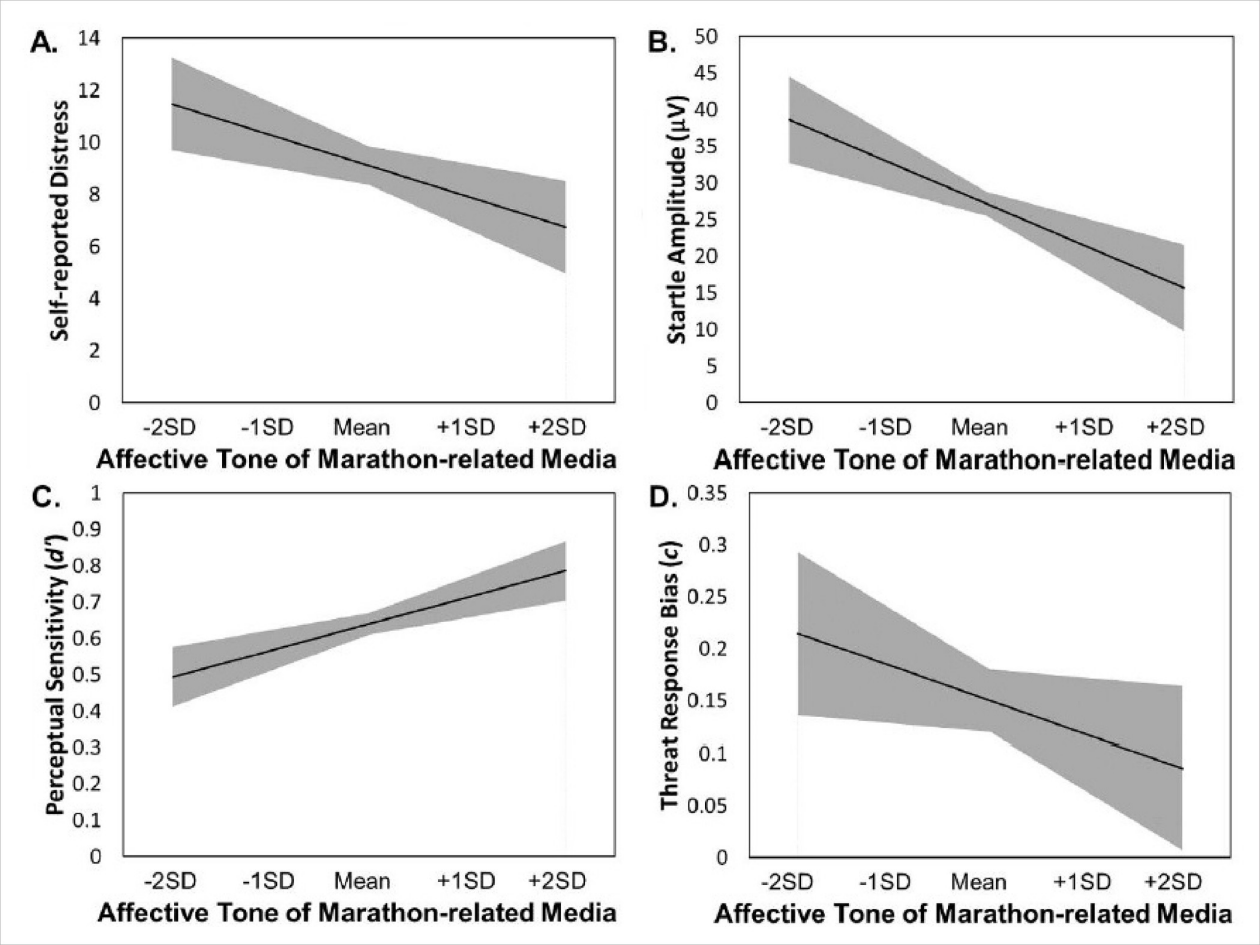
**Simple slopes representation of models predicting changes in distress (Panel A), startle reactivity (Panel B), perceptual sensitivity (Panel C), and threat response bias (Panel D) by changes in the affective tone of recent marathon-related coverage.** Affective tone of recent marathon-related media coverage was calculated to be time-locked for each participant at each Wave to include only media coverage from the two weeks prior to that participant's laboratory visit. Model slopes are represented by a black line, with ±1 standard error of the slope and intercept for each model shown as a gray-shaded area. SD stands for standard deviation. The model for perceptual sensitivity (Panel C) is plotted at the within-person mean for threat response bias, and the model for threat response bias (Panel D) is plotted at the within-person mean for perceptual sensitivity.

As reported in Table 2, at times when the marathon-related media coverage during the two weeks prior to a participant’s in-lab session was more affectively positive (v. negative), participants self-reported less distress related to the bombings (*B* = -28.26, *SE* = 12.42; *t*(90) = 2.28, *p* = .025; *d* = 0.48) and demonstrated decreased startle threat reactivity (*B* = -137.02, *SE* = 50.86; *t*(90) = 2.69, *p* = .008; *d* = 0.56). However, it should be noted that although the relationship with self-reported distress reached a traditional significance level of α = .05, it failed to reach a Bonferroni-corrected alpha of .0125 (to control for multiple comparisons). As hypothesized, more positive recent marathon-related media content also predicted increased perceptual sensitivity for discriminating threats from non-threats (*B* = 1.74, *SE* = 0.62; *t*(90) = 2.79, *p* = .006; *d* = 0.59) and was unrelated to response bias in the Shooter Bias Task (*B* = -0.77, *SE* = 0.58; *t*(90) = 1.33, *p* = .185; *d* = 0.28). Correspondingly, these findings also demonstrate that at times with more negative recent marathon-related content (relative to positive), participants exhibited significantly increased startle threat reactivity and self-reported distress related to the bombings, and showed significantly decreased perceptual sensitivity. Simple slopes representations of these models can be seen in Fig 1, and scatter plots and estimates of variance components are available in the supplemental online materials (See S2 Fig and S2 Table).

**Table 2:** Changes in affective tone of recent marathon-related coverage predicts distress, startle reactivity, perceptual sensitivity, and shooting behavior.

Outcome	B	SE	t-ratio	df	p	Cohen’s d
Self-reported Distress						
Model Intercept	9.11	0.74	12.25	90	< .001**	2.58
Model Slope	-28.26	12.42	2.28	90	.025*	0.48
Startle Amplitude						
Model Intercept	27.21	1.65	16.47	90	< .001**	3.47
Model Slope	-137.02	50.86	2.69	90	.008**	0.56
Perceptual Sensitivity						
Model Intercept	0.64	0.03	19.58	90	< .001**	4.12
Model Slope	1.74	0.62	2.79	90	.006**	0.59
Response Bias	0.38	0.08	5.02	90	< .001**	1.06
Threat Response Bias						
Model Intercept	0.15	0.03	4.78	90	< .001**	1.01
Model Slope	-0.77	0.58	1.33	90	0.185	0.28
P. Sensitivity	0.27	0.06	4.25	90	< .001**	0.9

Note: Higher affective tone values indicate more positive content while lower affective tone values indicate more negative content. Model uses robust standard errors (SE; i.e., random effects). Model coefficients (B) are unstandardized. Model Slope represents the coefficient estimates for the variable affective tone, which is centered around each participant’s own mean. Slopes can be interpreted as the predicted change in the outcome variable associated with a 1 unit increase in affective tone. For example, a participant’s startle amplitude is predicted to be 137.02 μV lower when there are an even number of positive and negative affective words in recent media coverage related to the Bombings than when all the affective words are negative.

*p < .05

**p < .0125 (Bonferroni-corrected alpha)

### Extent of recent marathon-related media coverage

**Fig 2:**
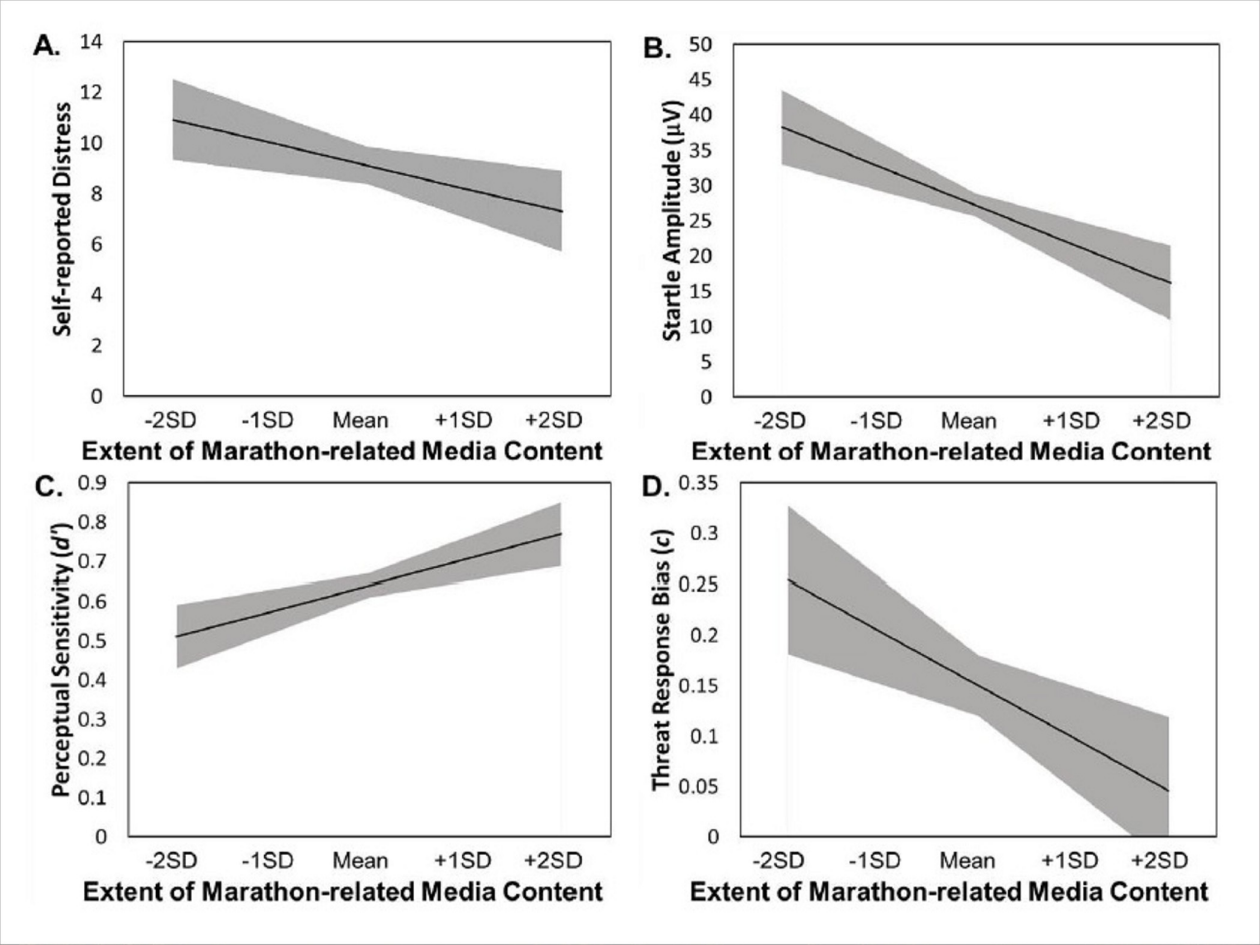
**Simple slopes representation of models predicting changes in distress (Panel A), startle reactivity (Panel B), perceptual sensitivity (Panel C), and Threat response bias (Panel D) by changes in the extent of recent marathon-related coverage.** Extent of recent marathon-related media coverage was calculated to be time-locked for each participant at each Wave to include only media coverage from the two weeks prior to that participant's laboratory visit. Model slopes are represented by a black line, with ±1 standard error of the slope and intercept for each model shown as a gray-shaded area. SD stands for standard deviation. The model for perceptual sensitivity (Panel C) is plotted at the within-person mean for threat response bias, and the model for threat response bias (Panel D) is plotted at the within-person mean for perceptual sensitivity.

As shown in Table 3, periods with a greater extent of recent marathon-related media coverage were associated with reduced self-reported distress related to the bombings (*B* = -7.13, *SE* = 3.41; *t*(90) = 2.09, *p* = .039; *d* = 0.44), reduced startle threat reactivity (*B* = -43.40, *SE* = 14.34; *t*(90) = 3.03, *p* = .003; *d* = 0.64), enhanced perceptual sensitivity for threat (*B* = 0.51, *SE* = 0.20; *t*(90) = 2.58, *p* = .011; *d* = 0.54), and a less conservative threat response bias (i.e., a lessened tendency to favor the “don’t shoot” response) (*B* = -0.41, *SE* = 0.17; *t*(90) = 2.45, *p* = .016; *d* = 0.52). However, it should be noted that whereas the relationships with self-reported distress and threat response bias reach a traditional significance level of α = .05, they fail to reach a Bonferroni-corrected alpha to control for multiple comparisons. Simple slopes representations of these models can be seen in Fig 2, and scatter plots and estimates of variance components are available in the supplemental online materials (see S3 Fig and S3 Table).

**Table 3:** Changes in extent of recent marathon-related coverage predicts distress, startle reactivity, perceptual sensitivity, and shooting behavior.

Outcome	B	SE	t-ratio	df	p	Cohen’s d
Self-reported Distress						
Model Intercept	9.11	0.74	12.23	90	< .001**	2.58
Model Slope	-7.13	3.41	2.09	90	.039*	0.44
Startle Amplitude						
Model Intercept	27.23	1.65	16.47	90	< .001*	3.47
Model Slope	-43.4	14.34	3.03	90	.003**	0.64
Perceptual Sensitivity						
Model Intercept	0.64	0.03	19.56	90	< .001**	4.12
Model Slope	0.51	0.2	2.58	90	.011**	0.54
Response Bias	0.39	0.08	5.12	90	< .001**	1.08
Threat Response Bias						
Model Intercept	0.15	0.03	4.78	90	< .001**	1.01
Model Slope	-0.41	0.17	2.45	90	.016*	0.52
P. Sensitivity	0.27	0.06	4.45	90	< .001**	0.94

Note: Model uses robust standard errors (i.e., random effects). Model coefficients (B) are unstandardized. Model Slope represents the coefficient estimates for the extent of recent marathon-related media content, which is centered around each participant’s own mean. Slopes can be interpreted as the predicted change in the outcome variable associated with a 1 unit increase in the extent of recent Marathon-related coverage. For example, a participant’s startle amplitude is predicted to be 43.40 μV lower when the likelihood of a reader seeing at least one marathon related article every day in the four newspapers assessed here is 100% than when it is 0%.

*p < .05

**p < .0125 (Bonferroni-corrected alpha)

## Discussion

Taken together, our findings demonstrate that people report more distress, exhibit increased startle reactivity, and are less perceptually sensitive at times when the affective tone of media coverage of an incident of mass violence is more negative (vs. more positive). Our findings are important because they show that self-report, physiological and behavioral outcomes are associated with differences in the tone of recent media coverage of mass violence, not solely the amount of media coverage. Past studies on media impact have demonstrated that people report more acute stress-related symptoms when they are exposed to more media coverage of an incident of mass violence [4, 5, 38] and show reduced perceptual sensitivity for threats [17, 18], but our findings suggest these relationships may be limited to media content that is more affectively negative in tone. In the present study, media coverage of a mass violence incident near its first anniversary was generally more positive in tone (this is apparent from Table 1, where the estimated mean for the affective tone of marathon-related media coverage at wave 2 is significantly higher than 0). In this context, we found that individuals reported *less* distress and were *more* perceptually sensitive to threats at times when there was more media coverage of the incident. These findings suggest that any potential impact of recent media coverage of an incident of mass violence may be moderated by whether that media coverage is more negative vs. more positive in affective tone (e.g., whether it tends to focus on the death and destruction caused by the event vs. the togetherness fostered in its aftermath).

Our finding that the affective tone of recent media coverage of an incident of mass violence is associated with concurrent changes in perceptual sensitivity for threats is an example of *affective realism*, the phenomenon whereby affective feelings contribute directly to one’s experience of the world [39–41]. From this theoretical perspective, feelings do more than influence impressions of what one has seen after the fact: they influence the actual content of perception. The human brain is not wired for perceiving the world objectively; it is wired to perceive the world in a way that is relevant to a person’s needs and well-being [42–44], and affect is infused into every perception and action as a result of the architecture of the nervous system [45–49]. Indeed, recent discoveries in neuroscience reveal that the human brain creates a unified conscious experience by integrating all sources of sensation, from both inside and outside the body, with limbic circuitry as the driver (for a discussion, see [45]). The present study extends this innovative neuroscience-based theory to the realm of threat perception, demonstrating that humans are *active* perceivers when it comes to threat and safety. People do not passively detect information in the world and then react to it–they actively construct perceptions of the world as the architects of their own experience [44, 45, 48].

In addition, the present study is novel in that we quantified the actual media content related to an incident of mass violence in the weeks immediately preceding each participant’s in-lab sessions, which represents a critical advance over prior work that has typically been limited to self-reports of media exposure. Here, instead of attempting to measure individual media exposure, we assessed media coverage more generally, capturing fluctuations in the average content and tone of recent written news media related to an incident of mass violence. Using this approach, we provide some of the first empirical evidence that the effects of media coverage of terrorist-related events may extend beyond self-reported outcomes to influence both physiological reactivity (specifically startle blink reactivity, which has been related to mental health outcomes following trauma exposure; [23]) and basic perceptual processing, such as identifying a handheld object as a weapon vs. an innocuous item. Despite these methodological advances, it is important to note that the present study is exploratory due to its novel approach, and results should be replicated in future confirmatory research prior to drawing any strong conclusions. In particular, the relationships between self-reported distress and the amount and tone of recent media coverage failed to reach a Bonferroni-corrected alpha level when controlling for multiple comparisons, and so should be subjected to rigorous scrutiny in future empirical work.

Interestingly, the news coverage of the Boston Marathon bombings near their first anniversary was generally positive in affective tone (see Table 1). Future research should examine whether this pattern is specific to the Boston Marathon bombings given their co-occurrence with a typically affectively positive, city-wide annual event (i.e., Patriots’ Day and the running of the Boston Marathon), or whether it reflects a more general trend whereby media coverage of mass violence incidents either (1) tends to be fairly neutral or even slightly positive even in early media reporting of the incidents (see, e.g., [49]) or (2) tends to become more positive as time passes since a mass violence incident. Given the importance of affective tone in determining how media coverage of mass violence is associated with changes in distress, physiological reactivity, and threat perception in the current study, identifying reliable patterns of change in the affective tone of such media content could have important translational value for interventions intended to minimize detrimental effects of community-wide media exposure to mass violence incidents. Moreover, future research should examine additional features of media coverage of mass violence incidents beyond affective tone, including specific emotional content or appraisals, to better understand when and how exposure to such coverage can be detrimental to viewers’ health and well-being. For instance, one study found that *fear of terror* in Israeli adults was one significant contributor to annual increases in resting heart rate, a notable risk factor for all-cause mortality [50].

Future research should also seek to extend findings beyond verbal content in newspapers to news media formats that are potentially more evocative, immersive, or pervasive (e.g., radio, television, social media). Cultivation theory, for instance, posits that television is fundamentally different from other forms of mass media and that greater television exposure can cause shifts in individuals’ perception of social reality, bringing perceived social reality more closely in line with reality as portrayed on television [51, 52]. In cultivation theory, effects of television exposure are assumed to be small, but pervasive: “just as an average temperature shift of a few degrees can lead to an ice age or the outcomes of elections can be determined by slight margins, so too can a relatively small but pervasive influences make a crucial difference. The “size” of an “effect” is far less critical than the direction of its steady contribution." ([52], p.14). Previous research examining how exposure to violence on television and/or in video games may increase aggressive or anti-social behavior [53, 54] has hypothesized similar underlying mechanisms concerning both the evocativeness of media content as well as its relentless ubiquity. Nevertheless, mounting empirical evidence has called into question the robustness of such effects on multiple grounds (see, e.g., [55, 56]). Consistent with these criticisms, the present study failed to find a reliable association between recent media coverage and threat response bias. While participants did tend to exhibit a less conservative response bias in our shooter bias task (i.e., a lessened tendency to favor the “don’t shoot” response) at times when there was a greater extent of media coverage of an incident of mass violence, this relationship failed to meet Bonferroni-corrected significance levels when controlling for multiple comparisons, and we found no relationship between threat response bias and the affective tone of media coverage of mass violence. Future work should examine whether relationships between media coverage and the more basic perceptual and physiological outcomes reported here are more robust than those involving more complex decision-making or behavior, such as exhibiting aggressive or anti-social behavior.

Finally, the present study may also offer a novel explanatory angle for consolidating seemingly contradictory findings related to whether repeated exposure to adversity or collective traumas leads to sensitization (e.g., [57–59]) or desensitization (e.g., [60–62]) to subsequent potential traumas (see also [63]). Our findings suggest that the affective responses to specific exposures may be critical in determining subsequent responses to similar potential threats or traumas. This logic is consistent with psychotherapy techniques in which the evocation of trauma-related emotional responses during therapy is a key feature of successful treatment. Specifically, prolonged exposure therapy (PET), a common evidence-based treatment for PTSD [64], involves repeated recall of a traumatic event over time. During treatment, clients are encouraged to maintain a high but tolerable level of distress during repeated exposures to the trauma narrative, in a process known as systematic desensitization. Our study suggests similar processes might be at play when repeated media coverage about a terrorist attack or incident of mass violence focuses less on the death and destruction caused by the attack and more on the strength and resilience of the affected communities in the wake of such tragedies.

## Supporting information

S1 FigMedia signaling indices for each outlet across the three waves.BG = Boston Globe, BH = Boston Herald, MT = Boston Metro, NY = New York Times. Volume refers to the total number of articles published per day, SR refers to the sentiment ratio (${\tilde{SR}}_{O}(t)$) for all articles, RR-mara refers to the relevance rate $\left({\tilde{RR}}_{O}(t)\right)$ for marathon-related articles, and SR-mara refers to the sentiment ratio $\left({\tilde{SR}}_{O}(t)\right)$ for marathon-related articles. All indices are smoothed with a 7-day sliding window as described in the main manuscript. Please note that the time scale depicted on the x-axis differs across waves.(DOCX)

S2 Fig Scatter plots for the affective tone of marathon-related content related to self-reported distress, startle amplitude, threat response bias, and perceptual sensitivity. In each plot, data points are not independent, as multiple data points are present for each of the 91 subjects included in analyses.
(DOCX)

S3 Fig Scatter plots for the extent of marathon-related content related to self-reported distress, startle amplitude, threat response bias, and perceptual sensitivity. In each plot, data points are not independent, as multiple data points are present for each of the 91 subjects included in analyses.
(DOCX)

S1 Table Summary of media data collection across waves. Articles refers to the number of articles collected from each outlet for each wave. Marathon-related articles refers to the number of articles containing the word “marathon” from each outlet for each wave. BG = Boston Globe, BH = Boston Herald, MT = Boston Metro, NY = New York Times.
(DOCX)

S2 Table Changes in affective tone of recent marathon-related coverage predicts distress, startle reactivity, perceptual sensitivity, and shooting behavior: Variance components. *r*_*0*_ and *r*_*1*_ refer, respectively, to the participant-level variability in the intercept and slope values (i.e., across-participant variability). *r*_*2*_ refers to the participant-level variability in the slope value for the control variable (i.e., bias or sensitivity) in models relating to threat perception. *e* refers to the estimated Level-1 error for each model (i.e., wave-level error). *p < .05
(DOCX)

S3 Table Changes in extent of recent marathon-related coverage predicts distress, startle reactivity, perceptual sensitivity, and shooting behavior: Variance components. *r*_*0*_ and *r*_*1*_ refer, respectively, to the participant-level variability in the intercept and slope values (i.e., across-participant variability). *r*_*2*_ refers to the participant-level variability in the slope value for the control variable (i.e., bias or sensitivity) in models relating to threat perception. *e* refers to the estimated Level-1 error for each model (i.e., Wave-level error). *p < .05
(DOCX)
