# Correction: Psychological impact of mass violence depends on affective tone of media content

**DOI:** 10.1371/journal.pone.0250756

**Published:** 2021-04-22

**Authors:** Jolie Baumann Wormwood, Yu-Ru Lin, Spencer K. Lynn, Lisa Feldman Barrett, Karen S. Quigley

After publication of the article [1], the authors notified the journal of errors in the text of the paper, and provided corrected figures, tables, and supporting information files. This paper reports a study that involved data collected at three time points. Due to a clerical error when merging data files, data collected during the final longitudinal time point (Wave 3) were assigned to the incorrect participants. The corrections do not impact the overall pattern of results. Please view the correct text, figures and tables below. Copies of the original and corrected data files are publicly available at https://osf.io/ym8hw/.

In the Questionnaires subsection of the Materials and methods, the following should be included as the second sentence: One participant did not complete the Wave 1 questionnaires and one participant did not complete the Wave 3 questionnaires.

In the Questionnaires subsection of the Materials and methods, there is an error in the second sentence of the fourth paragraph. The correct sentence is: Participants also completed a number of additional questionnaires unrelated to the current investigation, including measures of self-reported physical symptoms, depressive symptoms, anxiety symptoms, and neuroticism.

There is an error in the last sentence of the second paragraph of the Data processing subsection of the Materials and methods. This sentence should be removed from the paragraph:: For an analysis that examined differences in modulation of startle by picture type within this overarching affectively evocative context, see [29].

In the Data processing subsection of the Materials and methods, there is an error in the eighth sentence of the third paragraph. The correct sentence is: Shooter bias data for three participants at Wave 1 were omitted because their data was not saved due to computer error (*n* = 2) or because they did not follow task instructions (*n* = 1).

In the Affective tone of recent marathon-related media coverage subsection of the Results, there is an error in the first and third sentence of the first paragraph. The correct sentences are:

As reported in Table 2, at times when the marathon-related media coverage during the two weeks prior to a participant’s in-lab session was more affectively positive (v. negative), participants self-reported less distress related to the bombings (*B* = -26.02, *SE* = 8.65; *t*(90) = 3.01, *p* = .003; *d* = 0.63) and demonstrated decreased startle threat reactivity (*B* = -83.37, *SE* = 27.51; *t*(90) = 3.03, *p* = .003; *d* = 0.64).

As hypothesized, more positive recent marathon-related media content also predicted increased perceptual sensitivity for discriminating threats from non-threats (*B* = 1.44, *SE* = 0.62; *t*(90) = 2.31, *p* = .023; *d* = 0.49) and was unrelated to response bias in the Shooter Bias Task (*B* = -0.67, *SE* = 0.59; *t*(90) = 1.15, *p* = .255; *d* = 0.24).

In the Extent of recent marathon-related media coverage of the Results, there are errors in the first and second sentences of the first paragraph. The correct sentences are: As shown in Table 3, periods with a greater extent of recent marathon-related media coverage were associated with reduced self-reported distress related to the bombings (*B* = -8.14, *SE* = 2.50; *t*(90) = 3.25, *p* = .002; *d* = 0.69), reduced startle threat reactivity (*B* = -30.53, *SE* = 10.15; *t*(90) = 3.01, *p* = .003; *d* = 0.63), enhanced perceptual sensitivity for threat (*B* = 0.43, *SE* = 0.20; *t*(90) = 2.13, *p* = .036; *d* = 0.45), and a less conservative threat response bias (i.e., a lessened tendency to favor the “don’t shoot” response) (*B* = -0.44, *SE* = 0.17; *t*(90) = 2.59, *p* = .011; *d* = 0.55). However, it should be noted that whereas the relationship with perceptual sensitivity reached a traditional significance level of α = .05, it failed to reach a Bonferroni-corrected alpha to control for multiple comparisons.

In the Discussion, there is an error in the fifth sentence of the third paragraph. The correct sentence is: In particular, the relationships between perceptual sensitivity and the amount and tone of recent media coverage failed to reach a Bonferroni-corrected alpha level when controlling for multiple comparisons, and so should be subjected to rigorous scrutiny in future empirical work.

In the Discussion, there is an error in the eighth sentence of the fifth paragraph. The correct sentence is: While participants did tend to exhibit a less conservative response bias in our shooter bias task (i.e., a lessened tendency to favor the “don’t shoot” response) at times when there was a greater extent of media coverage of an incident of mass violence, we found no relationship between threat response bias and the affective tone of media coverage of mass violence.

Wormwood et al. was incorrectly included as reference 29. As a result, all subsequent references are misnumbered. References 30–64 should be references 29–63.

Figs 1 and 2 are incorrect. Please see the correct Figs 1 and 2 here.

**Fig 1:**
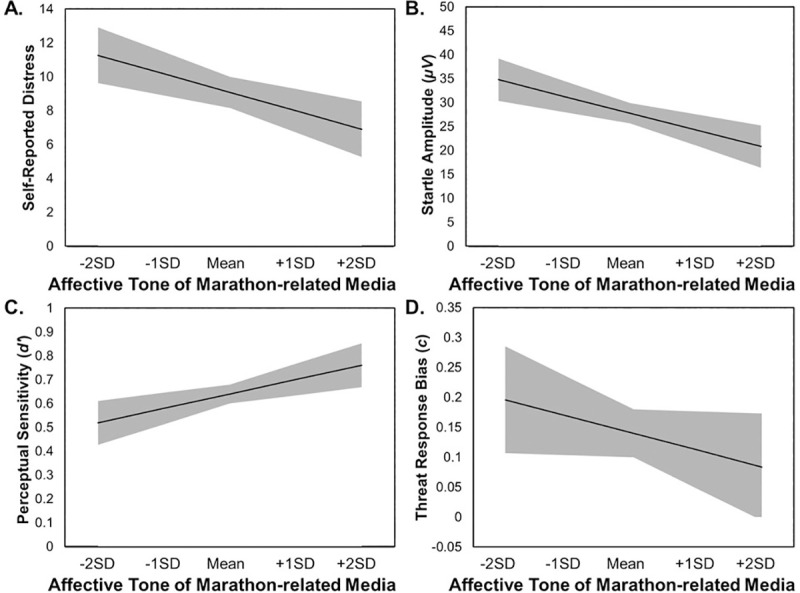
**Simple slopes representation of models predicting changes in distress (Panel A), startle reactivity (Panel B), perceptual sensitivity (Panel C), and threat response bias (Panel D) by changes in the affective tone of recent marathon-related coverage.** Affective tone of recent marathon-related media coverage was calculated to be time-locked for each participant at each Wave to include only media coverage from the two weeks prior to that participant’s laboratory visit. Model slopes are represented by a black line, with ±1 standard error of the slope and intercept for each model shown as a gray-shaded area. SD stands for standard deviation. The model for perceptual sensitivity (Panel C) is plotted at the within-person mean for threat response bias, and the model for threat response bias (Panel D) is plotted at the within-person mean for perceptual sensitivity.

**Fig 2:**
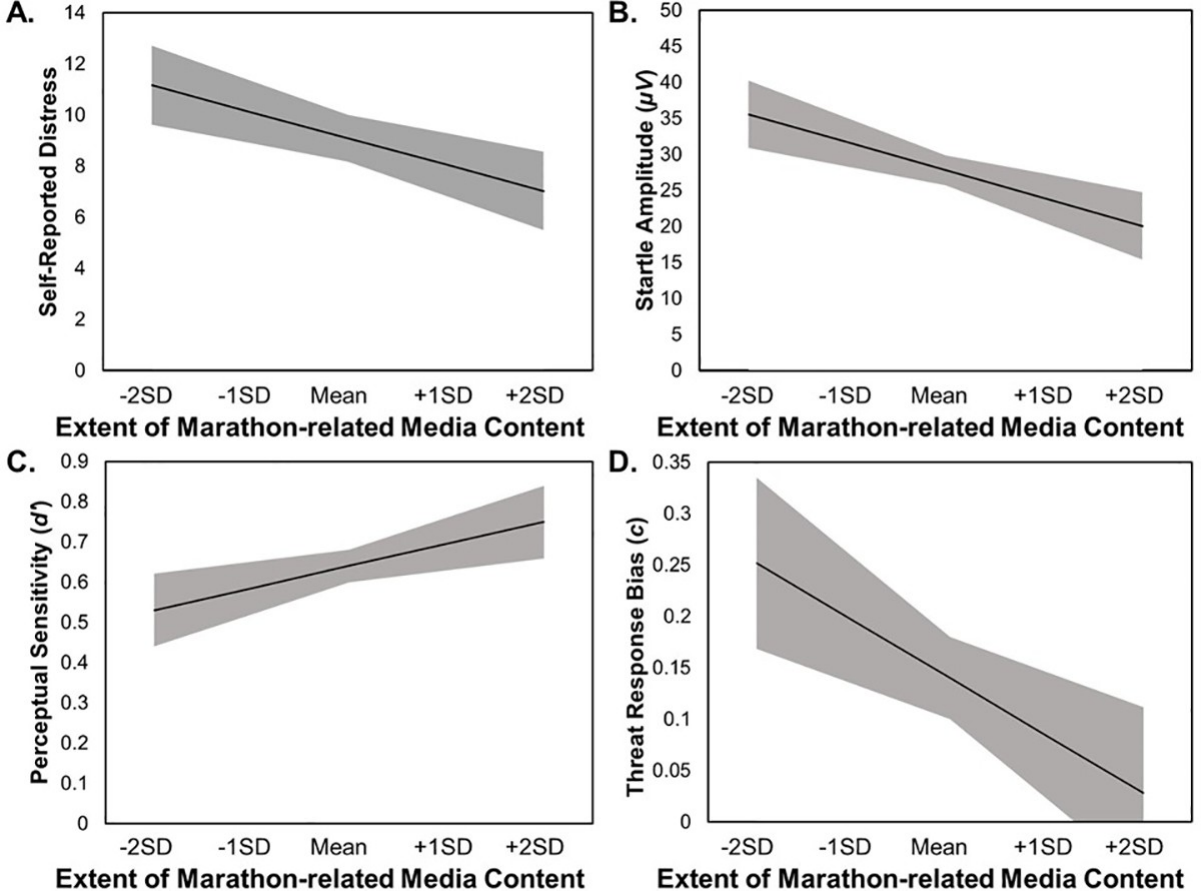
**Simple slopes representation of models predicting changes in distress (Panel A), startle reactivity (Panel B), perceptual sensitivity (Panel C), and Threat response bias (Panel D) by changes in the extent of recent marathon-related coverage.** Extent of recent marathon-related media coverage was calculated to be time-locked for each participant at each Wave to include only media coverage from the two weeks prior to that participant’s laboratory visit. Model slopes are represented by a black line, with ±1 standard error of the slope and intercept for each model shown as a gray-shaded area. SD stands for standard deviation. The model for perceptual sensitivity (Panel C) is plotted at the within-person mean for threat response bias, and the model for threat response bias (Panel D) is plotted at the within-person mean for perceptual sensitivity.

The values reported in Tables 1, 2 and 3 are incorrect. Please see the correct Tables 1, 2 and 3 here.

**Table 1:** Estimated Means and Standard Errors by Wave

Variable	Wave 1	Wave 2	Wave 3
Self-reported Distress	10.69 (1.06)^a^	9.16 (1.06)^b^	6.90 (0.94)^a,b^
Startle Amplitude	32.00 (3.13)^a^	20.81 (2.14)^a,b^	29.43 (2.86)^b^
Perceptual Sensitivity (*d’*)	0.55 (.05)^a^	0.69 (0.05)^a^	0.66 (0.05)
Threat Response Bias (*c*)	0.20 (0.04)^a^	0.05 (0.05)^a,b^	0.17 (0.04)^b^
Extent of Marathon-related Media Content (*Z*_*wi*_)	0.23 (0.01)^a^	0.49 (0.003)^a^	0.41 (0.01)^a^
Affective Tone of Marathon-related Media Content (*X*_*wi*_)	0.04 (0.004)^a^	0.10 (0.001)^a^	0.12 (0.003)^a^

Note: A slopes-as-intercepts approach in HLM was used to generate estimated means and standard errors (given in parentheses) for each variable across waves. Significant differences (α = .05, two-tailed) were determined using chi-square comparisons and are indicated by shared superscripts.

**Table 2:** Changes in Affective Tone of Recent Marathon-related Coverage Predicts Distress, Startle Reactivity, Perceptual Sensitivity, and Shooting Behavior.

Outcome	B	SE	t-ratio	df	p	Cohen’s d
Self-reported Distress						
Model Intercept	9.09	0.92	9.93	90	< .001**	2.10
Model Slope	-26.02	8.65	3.01	90	.003**	0.63
Startle Amplitude						
Model Intercept	27.80	2.11	13.21	90	< .001**	2.78
Model Slope	-83.37	27.51	3.03	90	.003**	0.64
Perceptual Sensitivity						
Model Intercept	0.64	0.04	16.50	90	< .001**	3.48
Model Slope	1.44	0.62	2.31	90	.023*	0.49
Response Bias	0.23	0.11	2.18	90	.023*	0.46
Threat Response Bias						
Model Intercept	0.14	0.04	3.77	90	< .001**	0.79
Model Slope	-0.67	0.59	1.15	90	0.255	0.24
P. Sensitivity	0.14	0.05	2.57	90	.012*	0.54

Note: Higher affective tone values indicate more positive content while lower affective tone values indicate more negative content. Model uses robust standard errors (SE; i.e., random effects). Model coefficients (B) are unstandardized. Model Slope represents the coefficient estimates for the variable affective tone, which is centered around each participant’s own mean. Slopes can be interpreted as the predicted change in the outcome variable associated with a 1 unit increase in affective tone. For example, a participant’s startle amplitude is predicted to be 83.37 µV lower when there are an even number of positive and negative affective words in recent media coverage related to the Bombings than when all the affective words are negative.

*p < .05

**p < .0125 (Bonferroni-corrected alpha)

**Table 3:** Changes in Extent of Recent Marathon-related Coverage Predicts Distress, Startle Reactivity, Perceptual Sensitivity, and Shooting Behavior.

Outcome	B	SE	t-ratio	df	p	Cohen’s d
Self-reported Distress						
Model Intercept	9.09	0.92	9.94	90	< .001**	2.11
Model Slope	-8.14	2.50	3.25	90	.002**	0.69
Startle Amplitude						
Model Intercept	27.81	2.10	13.21	90	< .001*	2.78
Model Slope	-30.53	10.15	3.01	90	.003**	0.63
Perceptual Sensitivity						
Model Intercept	0.64	0.04	16.51	90	< .001**	3.48
Model Slope	0.43	0.20	2.13	90	.036*	0.45
Response Bias	0.27	0.11	2.48	90	.015*	0.52
Threat Response Bias						
Model Intercept	0.14	0.04	3.77	90	< .001**	0.79
Model Slope	-0.44	0.17	2.59	90	.011**	0.55
P. Sensitivity	0.14	0.05	2.60	90	.011**	0.55

Note: Model uses robust standard errors (i.e., random effects). Model coefficients (B) are unstandardized. Model Slope represents the coefficient estimates for the extent of recent marathon-related media content, which is centered around each participant’s own mean. Slopes can be interpreted as the predicted change in the outcome variable associated with a 1 unit increase in the extent of recent Marathon-related coverage. For example, a participant’s startle amplitude is predicted to be 30.53 µV lower when the likelihood of a reader seeing at least one marathon related article every day in the four newspapers assessed here is 100% than when it is 0%.

*p < .05

**p < .0125 (Bonferroni-corrected alpha)

The scatter plots in S2 and S3 Figs are incorrect. Please view the correct S2 and S3 Figs below.

The values reported in S2 and S3 Tables are incorrect. Please view the correct S2 and S2 Tables below.

## Supporting information

S2 Fig Scatter plots for the affective tone of marathon-related content related to self-reported distress, startle amplitude, threat response bias, and perceptual sensitivity. In each plot, data points are not independent, as multiple data points are present for each of the 91 subjects included in analyses.
(DOCX)

S3 Fig Scatter plots for the extent of marathon-related content related to self-reported distress, startle amplitude, threat response bias, and perceptual sensitivity. In each plot, data points are not independent, as multiple data points are present for each of the 91 subjects included in analyses.
(DOCX)

S2 Table Changes in affective tone of recent marathon-related coverage predicts distress, startle reactivity, perceptual sensitivity, and shooting behavior: Variance components. *r*_*0*_ and *r*_*1*_ refer, respectively, to the participant-level variability in the intercept and slope values (i.e., across-participant variability). *r*_*2*_ refers to the participant-level variability in the slope value for the control variable (i.e., bias or sensitivity) in models relating to threat perception. *e* refers to the estimated Level-1 error for each model (i.e., wave-level error). *p < .05
(DOCX)

S3 Table Changes in extent of recent marathon-related coverage predicts distress, startle reactivity, perceptual sensitivity, and shooting behavior: Variance components. *r*_*0*_ and *r*_*1*_ refer, respectively, to the participant-level variability in the intercept and slope values (i.e., across-participant variability). *r*_*2*_ refers to the participant-level variability in the slope value for the control variable (i.e., bias or sensitivity) in models relating to threat perception. *e* refers to the estimated Level-1 error for each model (i.e., Wave-level error). *p < .05
(DOCX)
