# Supplementary material for: Psychological impact of mass violence depends on affective tone of media content
Source: PLoS One. 2019 Apr 1;14(4):e0213891. doi: 10.1371/journal.pone.0213891 (PMC6443148; doi:10.1371/journal.pone.0213891)
Supplement: S1 Fig — BG = Boston Globe, BH = Boston Herald, MT = Boston Metro, NY = New York Times. Volume refers to the total number of articles published per day, SR refers to the sentiment ratio (SR˜O(t)) for all articles, RR-mara refers to the relevance rate (RR˜O(t)) for marathon-related articles, and SR-mara refers to the sentiment ratio (SR˜O(t)) for marathon-related articles. All indices are smoothed with a 7-day sliding window as described in the main manuscript. Please note that the time scale depicted on the x-axis differs across waves. (DOCX) [file pone.0213891.s001.docx]

**S1 Fig. Media Signaling Indices for Each Outlet Across the Three Waves**


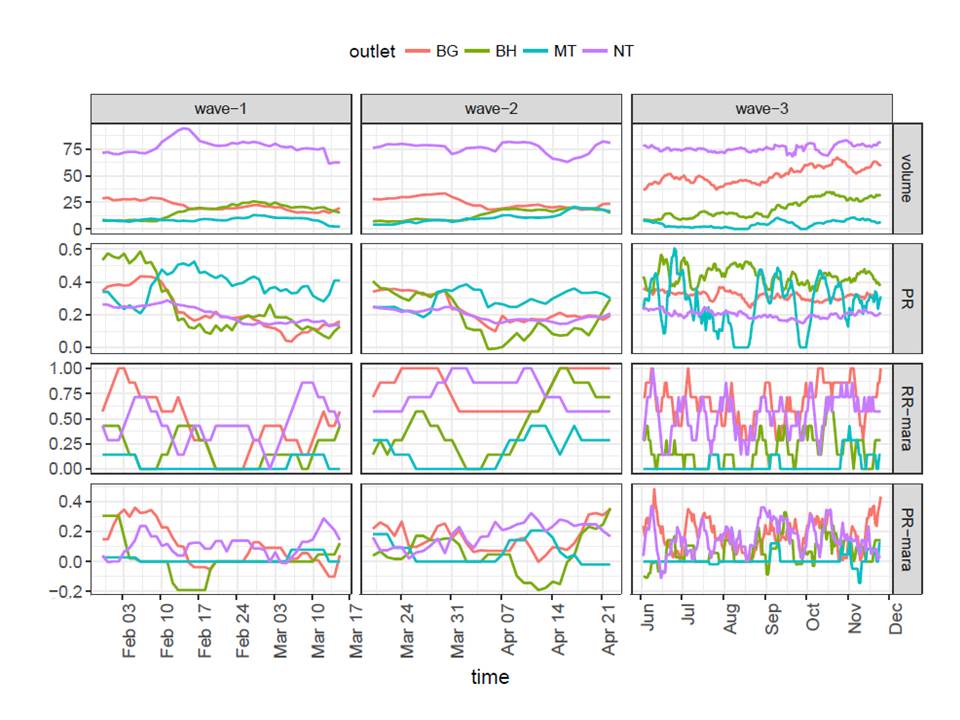


volume

RR-mara

SR-mara

SR

*Note:* BG=Boston Globe, BH=Boston Herald, MT=Boston Metro, NY=New York Times. Volume refers to the total number of articles published per day, SR refers to the positive sentiment ratio ($\tilde{SR}_{O}\left( t \right)$) for all articles, RR-mara refers to the relevance rate $\tilde{(RR}_{O}\left( t \right))$ for marathon-related articles, and SR-mara refers to the sentiment ratio $(\tilde{SR}_{O}\left( t \right))$ for marathon-related articles. All indices are smoothed with a 7-day sliding window as described in the main manuscript. Please note that the time scale depicted on the x-axis differs across waves.
