# Supplementary material for: Psychological impact of mass violence depends on affective tone of media content
Source: PLoS One. 2019 Apr 1;14(4):e0213891. doi: 10.1371/journal.pone.0213891 (PMC6443148; doi:10.1371/journal.pone.0213891)
Supplement: S3 Fig — In each plot, data points are not independent, as multiple data points are present for each of the 91 subjects included in analyses. (DOCX) [file pone.0213891.s003.docx]

**S3 Fig. Scatter Plots for the Extent of Marathon-related Content Related to Self-reported Distress, Startle Amplitude, Threat Response Bias, and Perceptual Sensitivity**

*Note:* In each plot, data points are not independent, as multiple data points are present for each of the 91 subjects included in analyses.
