# Supplementary material for: Psychological impact of mass violence depends on affective tone of media content
Source: PLoS One. 2019 Apr 1;14(4):e0213891. doi: 10.1371/journal.pone.0213891 (PMC6443148; doi:10.1371/journal.pone.0213891)
Supplement: S1 Table — Articles refers to the number of articles collected from each outlet for each wave. Marathon-related articles refers to the number of articles containing the word “marathon” from each outlet for each wave. BG = Boston Globe, BH = Boston Herald, MT = Boston Metro, NY = New York Times. (DOCX) [file pone.0213891.s004.docx]

**S1 Table. Summary of Media Data Collection Across Waves**

|  | In-Lab Data Collection Period | Media Data Collection Period | Articles | Marathon-related Articles |
| --- | --- | --- | --- | --- |
| **Wave 1** | 02/10/2014-03/11/2014 (30 days) | 01/24/2014-  03/12/2014 (48 days) | 6224 | 72 (1.16%) |
| BG |  |  | 1121 | 30 |
| BH |  |  | 823 | 10 |
| MT |  |  | 409 | 2 |
| NY |  |  | 3871 | 30 |
| **Wave 2** | 03/31/2014-04/19/2014 (20 days) | 03/14/2014-04/20/2014 (38 days) | 4942 | 173 (3.50%) |
| BG |  |  | 991 | 82 |
| BH |  |  | 513 | 43 |
| MT |  |  | 414 | 10 |
| NY |  |  | 3024 | 38 |
| **Wave 3** | 06/13/2014-11/21/2014 (162 days) | 05/27/2014-11/22/2014 (180 days) | 27343 | 380 (1.39%) |
| BG |  |  | 9161 | 193 |
| BH |  |  | 3534 | 41 |
| MT |  |  | 844 | 9 |
| NY |  |  | 13804 | 137 |

*Note:* Articles refers to the number of articles collected from each outlet for each wave. Marathon-related articles refers to the number of articles containing the word “marathon” from each outlet for each wave. BG=Boston Globe, BH=Boston Herald, MT=Boston Metro, NY=New York Times.
