# Supplementary material for: Psychological impact of mass violence depends on affective tone of media content
Source: PLoS One. 2019 Apr 1;14(4):e0213891. doi: 10.1371/journal.pone.0213891 (PMC6443148; doi:10.1371/journal.pone.0213891)
Supplement: S2 Table — r0 and r1 refer, respectively, to the participant-level variability in the intercept and slope values (i.e., across-participant variability). r2 refers to the participant-level variability in the slope value for the control variable (i.e., bias or sensitivity) in models relating to threat perception. e refers to the estimated Level-1 error for each model (i.e., wave-level error). *p < .05 (DOCX) [file pone.0213891.s005.docx]

**S2 Table. Changes in Affective Tone of Recent Marathon-related Coverage Predicts Distress, Startle Reactivity, Perceptual Sensitivity, and Shooting Behavior: Variance Components**

Outcome *SD* *Variance Component* *df* χ^2^ *p*

Self-Reported Distress

*r_0_* 5.05 25.47 88 173.50 <.001*

*r_1_* 34.66 1201.28 88 85.86 >.500

*e* 8.51 72.47

Startle Amplitude

*r_0_* 7.94 63.00 88 115.17 .019*

*r_1_* 258.13 66632.77 88 136.78 <.001*

*e* 22.35 499.50

Perceptual Sensitivity for Threat

*r_0_* 0.16 0.03 72 93.09 .048*

*r_1_* 0.24 0.06 72 69.44 >.500

*r_2_* 0.05 0.002 72 74.51 .396

*e* 0.45 0.20

Threat Response Bias

*r_0_* 0.21 0.04 72 151.99 <.001*

*r_1_* 0.95 0.89 72 86.84 .112

*r_2_* 0.14 0.02 72 98.51 .021*

*e*  0.37 0.13

*Note:* *r_0_* and *r_1_* refer, respectively, to the participant-level variability in the intercept and slope values (i.e., across-participant variability). *r_2_* refers to the participant-level variability in the slope value for the control variable (i.e., bias or sensitivity) in models relating to threat perception. *e* refers to the estimated Level-1 error for each model (i.e., wave-level error). **p*<.05
