# Supplementary material for: Psychological impact of mass violence depends on affective tone of media content
Source: PLoS One. 2019 Apr 1;14(4):e0213891. doi: 10.1371/journal.pone.0213891 (PMC6443148; doi:10.1371/journal.pone.0213891)
Supplement: S3 Table — r0 and r1 refer, respectively, to the participant-level variability in the intercept and slope values (i.e., across-participant variability). r2 refers to the participant-level variability in the slope value for the control variable (i.e., bias or sensitivity) in models relating to threat perception. e refers to the estimated Level-1 error for each model (i.e., Wave-level error). *p < .05 (DOCX) [file pone.0213891.s006.docx]

**S3 Table. Changes in Extent of Recent Marathon-related Coverage Predicts Distress, Startle Reactivity, Perceptual Sensitivity, and Shooting Behavior: Variance Components**

Outcome *SD* *Variance Component* *df* χ^2^ *p*

Self-Reported Distress

*r_0_* 4.90 23.98 88 165.71 <.001*

*r_1_* 3.08 9.51 88 60.29 >.500

*e* 8.71 75.87

Startle Amplitude

*r_0_* 7.14 50.99 87 105.11 .091

*r_1_* 58.39 3409.67 87 106.13 .080

*e* 23.40 547.44

Perceptual Sensitivity for Threat

*r_0_* 0.16 0.03 74 94.66 .053

*r_1_* 0.21 0.04 74 73.72 >.500

*r_2_* 0.04 0.001 74 83.96 .201

*e* 0.45 0.20

Threat Response Bias

*r_0_* 0.21 0.04 72 142.60 <.001*

*r_1_* 0.17 0.03 72 86.51 .117

*r_2_* 0.13 0.02 72 102.08 .011*

*e*  0.37 0.13

*Note:* *r_0_* and *r_1_* refer, respectively, to the participant-level variability in the intercept and slope values (i.e., across-participant variability). *r_2_* refers to the participant-level variability in the slope value for the control variable (i.e., bias or sensitivity) in models relating to threat perception. *e* refers to the estimated Level-1 error for each model (i.e., Wave-level error). **p*<.05
