# Supplementary material for: Correction: Psychological impact of mass violence depends on affective tone of media content
Source: PLoS One. 2021 Apr 22;16(4):e0250756. doi: 10.1371/journal.pone.0250756 (PMC8061993; doi:10.1371/journal.pone.0250756)
Supplement: S2 Table — r0 and r1 refer, respectively, to the participant-level variability in the intercept and slope values (i.e., across-participant variability). r2 refers to the participant-level variability in the slope value for the control variable (i.e., bias or sensitivity) in models relating to threat perception. e refers to the estimated Level-1 error for each model (i.e., wave-level error). *p < .05 (DOCX) [file pone.0250756.s003.docx]

**S2 Table. Changes in Affective Tone of Recent Marathon-related Coverage Predicts Distress, Startle Reactivity, Perceptual Sensitivity, and Shooting Behavior: Variance Components**

Outcome *SD* *Variance Component* *df* χ^2^ *p*

Self-Reported Distress

*r_0_* 8.07 65.17 87 557.22 <.001*

*r_1_* 6.06 36.73 87 102.11 .128

*e* 5.71 32.66

Startle Amplitude

*r_0_* 15.84 250.98 85 221.17 <.001*

*r_1_* 105.83 11200.73 85 51.40 >.500

*e* 20.39 415.86

Perceptual Sensitivity for Threat

*r_0_* 0.29 0.08 71 157.62 <.001*

*r_1_* 0.97 0.93 71 78.56 .252

*r_2_* 0.16 0.03 71 61.42 >.500

*e* 0.39 0.15

Threat Response Bias

*r_0_* 0.33 0.11 74 593.95 <.001*

*r_1_* 3.75 14.07 74 156.31 <.001*

*r_2_* 0.19 0.04 74 81.82 .249

*e*  0.21 0.05

*Note:* *r_0_* and *r_1_* refer, respectively, to the participant-level variability in the intercept and slope values (i.e., across-participant variability). *r_2_* refers to the participant-level variability in the slope value for the control variable (i.e., bias or sensitivity) in models relating to threat perception. *e* refers to the estimated Level-1 error for each model (i.e., wave-level error). **p*<.05
