# Supplementary material for: Correction: Psychological impact of mass violence depends on affective tone of media content
Source: PLoS One. 2021 Apr 22;16(4):e0250756. doi: 10.1371/journal.pone.0250756 (PMC8061993; doi:10.1371/journal.pone.0250756)
Supplement: S3 Table — r0 and r1 refer, respectively, to the participant-level variability in the intercept and slope values (i.e., across-participant variability). r2 refers to the participant-level variability in the slope value for the control variable (i.e., bias or sensitivity) in models relating to threat perception. e refers to the estimated Level-1 error for each model (i.e., Wave-level error). *p < .05 (DOCX) [file pone.0250756.s004.docx]

**S3 Table. Changes in Extent of Recent Marathon-related Coverage Predicts Distress, Startle Reactivity, Perceptual Sensitivity, and Shooting Behavior: Variance Components**

Outcome *SD* *Variance Component* *df* χ^2^ *p*

Self-Reported Distress

*r_0_* 8.07 65.15 87 558.61 <.001*

*r_1_* 3.05 9.29 87 77.69 >.500

*e* 5.71 32.57

Startle Amplitude

*r_0_* 16.25 264.07 86 241.56 <.001*

*r_1_* 46.95 2204.19 86 80.93 >.500

*e* 19.54 381.88

Perceptual Sensitivity for Threat

*r_0_* 0.29 0.08 75 169.60 <.001*

*r_1_* 0.41 0.17 75 81.96 .272

*r_2_* 0.14 0.02 75 77.58 .396

*e* 0.39 0.15

Threat Response Bias

*r_0_* 0.33 0.11 75 545.41 <.001*

*r_1_* 1.03 1.06 75 139.62 <.001*

*r_2_* 0.17 0.03 75 90.78 .104

*e*  0.22 0.05

*Note:* *r_0_* and *r_1_* refer, respectively, to the participant-level variability in the intercept and slope values (i.e., across-participant variability). *r_2_* refers to the participant-level variability in the slope value for the control variable (i.e., bias or sensitivity) in models relating to threat perception. *e* refers to the estimated Level-1 error for each model (i.e., Wave-level error). **p*<.05
